# The Naval Staff Question

**Published:** 1871-02

**Authors:** 


### Miscellaneous

### The Naval Staff Question.

There is just now a good deal of discussion at Washington, kept alive elsewhere by occasional articles in the daily papers, upon a subject which, considering its real importance, is surprisingly ill-understood—namely, the reorganization of the navy, more commonly called the "Staff Question." Mainly because the discussion relates to a "specialty," and is therefore gladly left for argument and settlement to those specially concerned, and partly because of the ill-advised efforts of too zealous advocates who rush into print on either side, the real matter at issue has been lost sight of in a fog of side-issues and technicalities, until the interested public has been narrowed down to the small number whose relatives and friends are directly affected by the present status of the navy, which a part of them desires to change. Yet, if it is worth while to have a navy at all, it is worth while to see that it be efficient; and the smaller the number of ships and officers to which it is reduced, the more important becomes the state of efficiency and readiness in which it is maintained. No doubt, small quarrels and petty conflicts of authority and precedence will inevitably and frequently occur among gentlemen whose bile is daily stirred up and temper soured by the discomforts and confinement of sea voyages, and the public has very sensibly ceased to take much interest in naval squabbles. But the recent dispute really appears tobe rooted too deeply to be lightly disregarded. On shore, as well as at sea, and during more than two years, the division has been growing wider and deeper; and is persistently alleged that, unless certain points are settled definitely by law, and no longer left to the elastic rules of usage and precedent, the efficiency of the service willbe seriously impaired, while its internal harmony can now scarcely be said to exist at all. These points of issue are between the line officers and those of the staff, the latter being inclusive of surgeons, paymasters and engineers; and can scarcely be made intelligible without a brief explanation.

The functionaries of a man-of-war are, first, the commanding officer; second, an "executive officer," or first lieutenant, who *executes* the order of the commander, and should not originate any orders himself. This officer presides in the ward-room, and is supposed to exercise general supervision over all departments of the ship. Officers desiring to leave the ship or to communicate with the captain, whatever their nominal rank or length of service, must first ask the "executive's" permission; all reports to the captain must be first represented to him; and, in fine, he stands between the commander and all others on board, and, by special regulation, takes precedence of all staff-officers. Next in rank among line officers is the navigator, who performs the duties indicated by his title, which on merchant vessels belong to the captain; and below him are four or more watch-officers, who take charge of the deck in turn for four hours at a time. Of the staff there are a surgeon, paymaster and engineer, and on large vessels an assistant surgeon and one or more assistant engineers.

In support of the present condition of affairs, the line officers contend that effi ciency equires rigid discipline, which implies absolute, irresponsihle command on the one hand, and unqualified obediance on the other; that such power of command must reside in the captain and in his representatives, whatever their nominal rank; and that such representatives are the executive officer and the officer of the deck for the time being. Should staff-officers be allowed actual rank—say the line—circumstances might frequently arise in which they would be entitled to command the ship, a sphere of duty for which they are totally unfitted by education. Moreover, they maintain that, for the proper maintenance of discipline, a superiority of their own, as the governing class and essentially *the navy*, must be acknowledged, particularly by those whom they delight in calling "the auxiliary officers" of the navy.

The staff, on the other hand, claim to understand best the details of the management of their several departments, and state that, under the present system, the efficiency of the service is frequently and seriously impaired by unwarrantable and petty interferences on the part of young and subordinate line officers. They therefore ask for the control of their respective departments, subject only, though entirely, to the captain of the ship. They demand, to this end, and as the only practical remedy, actual rank (but expressly disclaim the right of command in the line, or outside of their several specialties) and the right to quarters in the cabin. With this rank they ask for the dignities, immunities, and privileges which it conveys to the line, with the exceptions above mentioned; and that the precedence of the executive officers over themselves be limited, as in the line, to cases in which he is senior in lineal rank, or by date of commission. These demands have been embodied in the Stevens Bill, which is now pending in the Senate.

The result of the present state of uncertainty is certainly bad, and calls for a remedy. The naval service has become a house divided against itself, to the point of almost absolute non-intercourse. Officers go to sea bristling with jealous watchfulness of each other's actions, and ready constantly to seize upon the first pretext for a dispute. Reports, based often upon the most trivial grounds, are more frequent than in a young ladies' boarding-school; and, from such a state of feeling, delays, want of *esprit de corps*, and occasionally positive public damage have resulted. In the medical corps, for example, although an examining board is constantly in session, there are to-day over fifty vacancies, and when, in a profession notoriously so overcrowded as the medical, not so many can be found able to pass the moderate examination, and willing to accept a position which should be honorable, there follows a strong presumption that the alleged injustice to staff-officers has good foundation in fact. The medical profession, indeed, has taken up the cudgels actively, and there is now scarcely a medical association in the country which has not passed resolutions calling for legislation in this matter, and, so far, discouraging capable physicians from offering themselves as doctors in our national vessels.

In support of their assertion, that actual rank will prove a sufficient remedy for the hardships and hindrances which they suffer, the staff-officers point to the well-known success of the staff organization in the army, where the provisions of the Stevens Bill have been long in practical operation. The efficiency of the Army Medical Department has really been a wonder to the scientific world in general. Its circulars are accepted as the best of authorities in Europe as well as at home, and it has become a legitimate source of pride to every American who know its history and values the true honor of his country. If this great success and acknowledged superiority be, as alleged, the result of independence of action and of freedom from the control of those not experts in medical and surgical matters, doubtless the instance is well chosen and applicable.

Or take the case of the paymasters: the cost to the Government of disbursing its money, including the pay of officers transportation and *defalcations*—much noise has been made about this and that notorious case of embezzlement—was less than one-sixteenth of one per cent.—a fact showing, as the advocates of the Civil Service Bill have well said, that the surest protection to the Government against the dishonesty of its officials is to be found by making its offices permanent and respectable, thereby attracting a class of men so high as to be above the commoner temptations to fraud. Concerning the army system, Gen. Sherman writes to Admiral Porter, that it "works very well in practice," and such is the testimony of army officers generally.

It appears, also, that in other counties this distinction which staff-officers find it so hard to endure does not exist. In the Russian navy, for exampie, they attain to the highest rank [general admiral,] in the British and Spanish to that of vice-admiral; in the French and Austrian to rear-admiral, without impairing either efficiency or discipline.

There are at least two facts to be deduced from the mass of contradictory statements on both sides of this quarrel. One is, that the staff-officers of the navy are quite convinced that they are unjustly treated, and are clear as to the remedy: and the other is, that this remedy has the merit of being no new or untried experiment, but a plan which has worked well in practice; in the United States army and in the navies of other countries. Its merits must be decided by Congress, since every effort to settle the question by means of mixed boards, and by reference to those most thoroughly cognizant of the circumstances, has resulted in a strictly party division and no agreement upon essential points. Certainly the picture which has been presented of the aged fleet surgeon or paymaster asking the executive officer, not born when he entered the service, "for permission to go on shore," reporting to the beardless ensign pacing the quarter-deck that he has "permission to leave the ship," and then waiting for a still lesser younger to take command of the boat which is to convey him ashore, manifests an inherent absurdity which is yet the necessary result of existing laws and regulations. Or think of a competent surgeon condemned by a naval court-martial for declining to take a man off the sick list and declaring him too ill for duty, when a certain line officer—who had disabled the man by punishment—demanded that the surgeon should report him well.

The subject is of more importance than it seems, for few of those who remain at home are aware to how great an extend foreign ideas of Americans, particularly outside of Europe, are based upon the demeanor, attainments, and ability of the officers of the navy. Aside from the undoubted necessities of war, it is a mater of considerable importance to this country that, at least until the diplomatic service can be set upon a more creditable footing, the navy should be so constructed and directed as to attract the best attainable material into both staff and line corps. And in no way can this be better effected than by at least equalizing the status of professionable men on board ship with that which they would occupy in the military service on shore.

Much, if not most, of the tronble now existing is to be attributed to the introduction of equivocal terms, such as "assimilated rank," admitting of various constructions according to the whim or prejudice of digerent officers, and it is to be hoped that the subject will not only be fully discussod in Congress, but finally settled by a plain and intelligible enactment.—*The Nation.*

